# Social Inequalities in Adolescents’ Psychological and Somatic Complaints: Cross-National Trends Between 2002 and 2022 and the Role of Societal Changes

**DOI:** 10.3389/ijph.2024.1607709

**Published:** 2025-01-31

**Authors:** Mathilde E. Brons, Paola Berchialla, Marco Helbich, Maxim Dierckens, Michela Lenzi, Joanna C. Inchley, Gonneke W. J. M. Stevens

**Affiliations:** ^1^ Department of Interdisciplinary Social Science, Utrecht University, Utrecht, Netherlands; ^2^ Department of Clinical and Biological Sciences, University of Torino, Torino, Italy; ^3^ Department of Human Geography and Spatial Planning, Utrecht University, Utrecht, Netherlands; ^4^ Department of Public Health and Primary Care, Ghent University, Ghent, Belgium; ^5^ Department of Developmental and Social Psychology, University of Padova, Padova, Italy; ^6^ MRC/CSO Social and Public Health Sciences Unit, University of Glasgow, Glasgow, United Kingdom

**Keywords:** adolescents’ psychological and somatic complaints, family SES, social inequalities, cross-national trends, HBSC, income inequality, schoolwork pressure, internet activity

## Abstract

**Objectives:**

Cross-national differences in long-term trends in social inequalities in adolescents’ mental health remain poorly understood, as does the impact of societal changes. We tested (1) whether the association between family socioeconomic status and psychological and somatic complaints changed between 2002 and 2022, (2) the extent to which these trends varied across countries, and (3) whether changes in income inequality, schoolwork pressure, and internet activity within countries were related to these trends.

**Methods:**

Using data from 903,344 adolescents across 32 countries from the Health Behaviour in School-aged Children (HBSC) study between 2002 and 2022, we employed multilevel models to investigate the research questions.

**Results:**

We observed a nonlinear increase in psychological and somatic complaints over time. On average, social inequalities in both outcomes remained stable across countries, although the trends varied from one country to another. Only income inequality explained the differences between countries in these trends. In countries where income inequality increased over time, social inequalities in psychological complaints became smaller.

**Conclusion:**

Our study highlights ongoing global disparities in adolescents’ mental health problems, urging for more effective health policies.

## Introduction

Numerous studies in different European countries have shown social inequalities in adolescents’ mental health problems [1–3], with adolescents from families with a lower socioeconomic status (SES) consistently reporting more mental health problems. In this paper, we define social inequalities in adolescents’ mental health problems as the unequal distribution in mental health problems across different social groups – a concept also referred to as social gradient – measured here by family affluence [4, 5]. Some studies suggest that the association between family SES and adolescents’ mental health problems may have become more pronounced over time, given the increase in stressors stemming from societal changes like more pressure to perform academically [6], more social comparisons facilitated by social media [7], and a more pronounced social class competition [8, 9]. These societal changes may foster an environment where adolescents are more likely to compare themselves to their peers, which can lead to heightened pressure to perform as well as to stress. Although these societal changes seem to have adverse effects on the mental health of all adolescents [8], they may be more harmful to those with a low family SES background [9]. Low SES adolescents are more likely to experience stressors from the family domain (e.g., parental conflict) [2] and to experience feelings of inferiority deriving from their status; thus, when encountering stressors from societal changes fostering social comparison and competitive processes (such as school pressure and internet activity), it may become too much for them. Consequently, this cumulative disadvantage for adolescents from lower SES families can disproportionately impact their mental health problems [10, 11], potentially widening social inequalities in adolescents’ mental health problems over time.

Relatively little is known about international trends in social inequalities in adolescent mental health problems. Most studies have been conducted within *single countries*, revealing contrasting results. To illustrate, a Scottish study between 1998 and 2018 [12] and one in Sweden between 2004 and 2020 [13] showed widening social inequalities in adolescents’ mental health problems, whereas studies in the Netherlands (2001–2017) [14] and Norway (2014–2018) [7] indicated stable trends. *Cross-national studies* either demonstrated temporally stable associations between family SES and adolescents’ mental health problems across countries [15–17] or increasing social inequalities in mental health problems for psychological complaints and stable ones for somatic complaints [18]. These studies underscore country differences in trends of social inequalities in mental health problems. However, these studies assessed trends up until 2014, potentially not taking into account more recent societal changes impacting social inequalities in adolescents’ mental health problems [18].

Except for Elgar et al. [9], these cross-national studies did not explore which societal changes contribute to shifts in time trends in social inequalities in adolescents’ mental health problems, nor did they explain the cross-national variation in these trends. Elgar et al. [9] demonstrated that in countries where income inequality increased, social inequalities in psychological complaints increased over time. Our analysis extends beyond economic changes to investigate changes in income inequality, schoolwork pressure, and social media use at the country level as potential explanatory factors for cross-national differences in trends in social inequalities in adolescents’ mental health problems. As mentioned above, these societal changes may be more detrimental for low SES adolescents due to the combination of stressors inherent to their SES [19], possibly widening social inequalities over time. Although prior studies observed increases in stressors stemming from societal changes over time, particularly schoolwork pressure and social media use, in many countries, the extent of these increases varies considerably across countries [18, 20, 21], potentially accounting for the cross-national variation in trends in social inequalities in adolescents’ mental health problems. Hence, we examined trends in social inequalities in adolescents’ psychological and somatic complaints across 32 countries between 2002 and 2022. Using data from the international Health Behaviour in School-aged Children (HBSC) study, three research questions were addressed:

1. To what extent do social inequalities in adolescents’ psychological and somatic complaints change over time on average across 32 countries?
2. To what extent do these trends in social inequalities in adolescents’ psychological and somatic complaints vary across countries?
3. To what extent are changes in income inequality, schoolwork pressure, and social media use within countries related to trends in social inequalities in adolescents’ psychological and somatic complaints?

We expected widening social inequalities in adolescents’ psychological and somatic complaints over time. However, considering differences in societal changes between countries, we also hypothesized variations in the trends in social inequalities in adolescents’ psychological and somatic complaints across countries. Specifically, we expected that widening social inequalities in adolescents’ psychological and somatic complaints would be particularly prevalent in countries with greater over-time increases in income inequality, schoolwork pressure, or social media use.

## Methods

### Data

The main data source for the present study is the Health Behaviour in School-aged Children (HBSC) study. This cross-sectional study includes a large representative school-based survey conducted every 4 years since 1983 in collaboration with the World Health Organization (WHO) Regional Office for Europe. HBSC monitors health behaviour and wellbeing of adolescents aged 11, 13, and 15 across 49 countries/regions in Europe, Canada, and Israel, including sub-national information for Belgium (Flanders and Wallonia) and Great Britain (England, Scotland, and Wales). We refer to all countries and regions collectively as “countries” in the remainder of this article. All participating countries adhered to a standard international protocol to ensure consistency of measures, sampling, and implementation procedures [22]. Institutional ethical approval was obtained in each country.

For our analysis, we focused on survey cycles from 2002 to 2022. Only countries participating in at least three out of the six survey cycles were retained to assess changes over time, as this minimum is necessary to reliably capture trends and fluctuations across different time periods (i.e., *n*_*individuals*_ = 1,224,209; *n*_*countries*_ = 43) [23]. After excluding adolescents with missing data on individual- or country-level variables, our final sample comprised 903,344 adolescents (female: 50.58%; *M*_*age*_ = 13.56; *SD*_*age*_ = 1.64) from 32 countries. The excluded participants did not differ significantly from the retained sample in terms of psychological and somatic complaints, gender, age, family structure or family SES.

### Measures

#### Outcome Variables

Adolescents’ mental health problems were measured as *psychological* and *somatic complaints*. Both types of complaints were measured with the HBSC Symptom Checklist [24] assessing the frequency of four psychological (feeling low, irritable/bad tempered, feeling nervous, and having trouble sleeping) and four somatic symptoms (headache, stomachache, backache, and feeling dizzy) experienced over the past 6 months. Each symptom was assessed using a five-point-response scale ranging from “about every day” (1) to “rarely or never” (5). Scores were averaged after reverse-coding items for adolescents who completed at least two items per subscale, with higher values referring to more psychological and somatic complaints. Earlier studies demonstrated the validity and reliability of these two subscales across countries and their strong correlations with other mental health indicators such as emotional problems (r = −0.79, p < 0.001) [25, 26].

#### Independent Variables

*Family SES* was measured at the individual level using the Family Affluence Scale (FAS) capturing families’ material assets [27, 28]. Initially consisting of four items (own bedroom, number of cars, computers, and holidays), two additional items (bathroom and dishwasher) were introduced from 2010 onwards [29]. FAS sum scores were standardized per survey year and country for comparability and adjusted for varying economic conditions across countries and times using ridit-transformation [30, 31]. These ridit scores reflect the proportion of respondents with lower family affluence within countries and years. The mean for each age group, country, and survey year was set to 0.5 to ensure that half of the respondents within a country and year fell below and half above the mean level of family affluence.

Country-level *income inequality* was measured through the post-taxation Gini index per corresponding survey year and country obtained from the Standardized World Income Inequality Database [32], as this information is not available through HBSC. The Gini index quantifies the extent to which the income distribution among individuals or households deviates from an equal distribution within an economy ranging from 0 to 1, with higher scores denoting more country-level income inequality.

Country-level *schoolwork pressure* was measured by the question: “How pressured do you feel by the schoolwork you have to do?”. Participants responded on a four-point scale ranging from “not at all” (1) to “a lot” (4). We computed the average amount of schoolwork pressure per survey year within each country, with higher values referring to more schoolwork pressure. This measure has been frequently used in cross-national studies [18, 33, 34].

As a proxy for country-level social media use, we used data on *internet activity* derived from the PISA study 2012, 2015, 2018, and 2022, since HBSC only included the social media item from 2018 onwards. Until 2018, respondents were asked about their typical weekday internet usage outside of school [35]. A modification to the question was made in 2022 including various activities both inside and outside the school such as playing video games and reading/listening to informational materials [36]. To ensure consistency over time, we focused on after-school activities and coded responses to reflect daily hours: no time = 0, 1–30 min per day = 0.25, 31–60 min per day = 0.75, between 1 and 2 h per day = 1.5, between 2 and 4 h per day = 3, between 4 and 6 h per day = 5, more than 6 h per day = 7. Subsequently, the average hours spent on internet use per country and survey year were computed. Due to non-parallel survey cycles of HBSC and PISA, HBSC data were linked to the closest PISA data, with a maximum difference of 2 years between the two surveys.

#### Control Variables

We controlled for *age* (in years), *gender* (0 = boy, 1 = girl), and *family structure* (i.e., whether (0) or not (1) adolescents lived together with both parents in the primary household). On the country level, we included *social welfare spending* measured as a percentage of the gross domestic product (GDP) from the OECD [37] and wealth measured as gross national income (GNI) for each country and year from the World Bank [38]. Also, we controlled for country means across years of income inequality, schoolwork pressure, and internet activity to isolate the within-country effects [39]. For instance, while certain countries may generally exhibit high levels of schoolwork pressure regardless of societal changes, this method enables us to identify whether changes in schoolwork pressure may be attributable to varying trends.

### Analytical Strategy

We employed multilevel models to assess the associations between family SES and either psychological or somatic complaints, acknowledging the nested structure of adolescents within country-years within countries. The school level was excluded due to convergence issues. A random slope captured varying effects across countries of the interaction term with time and family SES. B-splines - piecewise polynomial functions that enable local control over curve smoothness - were employed to model linear, quadratic, and cubic changes over time [40], allowing for greater flexibility in capturing complex, (non-)linear trends. The B-splines modeled time-related changes more accurately than conventional linear models. We combined these three forms of changes (i.e., linear, quadratic and cubic changes) into our time variable in our models.

For our main analyses, three models per outcome were fitted. A *null model* (i.e., model without independent variables) determined the intraclass correlation coefficient (ICC) per level (i.e., individual, country-years, and country) [41]. To assess if the association between family SES and psychological and somatic complaints changed over time, *Model 1* included a cross-level interaction between time and family SES. *Models 2a-c* separately included a three-way cross-level interaction between respectively income inequality (*Model 2a*), schoolwork pressure (*Model 2b*), and internet activity (*Model 2c*) with family SES and time to examine if societal changes in country-level independent variables contributed to shifts in trends regarding social inequalities in psychological and somatic complaints, focusing on within-country effects. We used two-way interactions to explore if higher values of country-level variables, either through comparisons between countries or over time, were associated with greater social inequalities, capturing both between– and within-country effects. We excluded the three-way interactions to allow a meaningful interpretation of the two-way interactions.

For the cross-level interactions, we grand-mean centered family SES, adjusting it per country and year. Likewise, country-level income inequality, schoolwork pressure, and internet activity were centered per country [36]. Sample sizes varied across models based on the data availability at the country-year level. All analyses were conducted in R 4.2.3 [42] using the packages “lme4” [43] and “lmerTest” [44].

## Results

Descriptive statistics are detailed in Table 1, and bivariate correlations are in the Supplementary Appendices (Supplementary Table SA1). No multicollinearity was detected, as all VIF values remained below the critical value of 10 [45] (Supplementary Table SA2).

**TABLE 1:** Descriptive statistics of the study variables between 2002–2022 (Health Behaviour in School-aged Children, N_individuals_ = 902,682, N_countries_ = 32).

Variables	Min	Max	*2002*	*2006*	*2010*	*2014*	*2018*	*2022*
			*N*_*individuals*_ *= 116,276*	*N*_*individuals*_ *=147,039*	*N*_*individuals*_ *=159,094*	*N*_*individuals*_ *= 152,737*	*N*_*individuals*_ *= 158,205*	*N*_*individuals*_ *= 169,331*
			Mean (SD)/%	Mean (SD)/%	Mean (SD)/%	Mean (SD)/%	Mean (SD)/%	Mean (SD)/%
**Outcome variables**								
Psychological complaints	1	5	2.18 (0.94)	2.18 (0.97)	2.17 (0.97)	2.24 (1.02)	2.36 (1.04)	2.68 (1.13)
Somatic complaints	1	5	1.79 (0.80)	1.79 (0.93)	1.82 (0.84)	1.85 (0.86)	1.84 (0.85)	2.06 (0.96)
**Individual-level variables**								
Gender (*ref. = boys*)	0	1	51.79%	51.54%	51.60%	51.61%	51.42%	50.87%
Age	11	15	13.54 (1.66)	13.60 (1.65)	13.57 (1.64)	13.63 (1.62)	13.53 (1.61)	13.67 (1.64)
Family structure (*ref. = not living together with both parents)*	0	1	77.63%	74.02%	73.43%	73.43%	72.15%	75.99%
Family SES	0	1	0.50 (0.28)	0.50 (0.28)	0.50 (0.28)	0.50 (0.29)	0.50 (0.29)	0.50 (0.28)
**Country-level variables**								
Income inequality	0.22	0.38	0.29 (0.04)	0.30 (0.04)	0.30 (0.04)	0.30 (0.03)	0.29 (0.03)	0.29 (0.03)
Schoolwork pressure	1.84	2.83	2.26 (0.21)	2.27 (0.20)	2.22 (0.20)	2.27 (0.18)	2.34 (0.22)	2.51 (0.17)
Internet activity	1.73	5.33			2.54 (0.34)	3.28 (0.35)	3.73 (0.30)	4.75 (0.35)
GNI	0.00	0.42	0.05 (0.07)	0.08 (0.10)	0.07 (0.09)	0.10 (0.12)	0.09 (0.11)	0.10 (0.12)
Social welfare	0.12	0.32	0.20 (0.04)	0.20 (0.05)	0.23 (0.04)	0.23 (0.05)	0.22 (0.05)	0.23 (0.05)

*Note*: all variables are uncentered for descriptive statistics; internet activity was not available for the survey cycles 2002 and 2006; the number of countries varied given the different availability of the country-level variables; descriptive statistics employed listwise deletion for missing data (incl. psychological and somatic complaints).

### Model Verification

**FIGURE 1:**
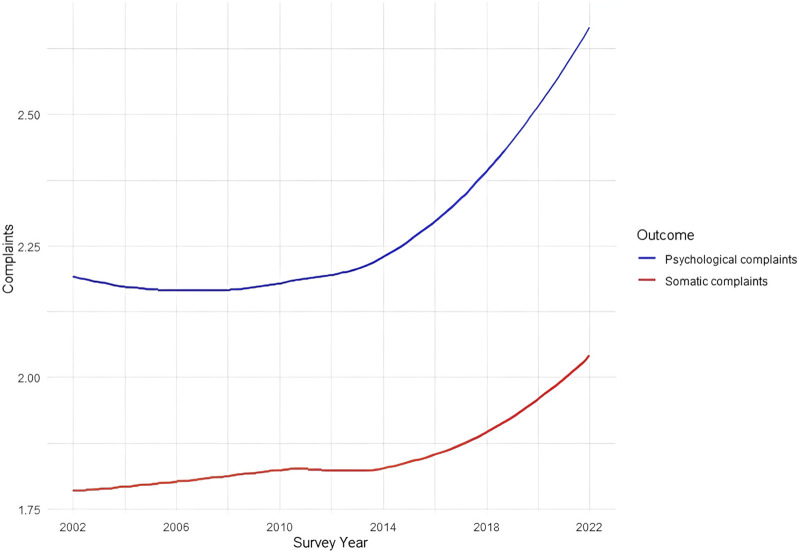
Trends in psychological and somatic complaints between 2002–2022 (Health Behaviour in School-aged Children, N_individuals_ = 902,682, N_countries_ = 32). Note: The scale ranges between 1 and 5 scores, with higher scores referring to more somatic or psychological symptoms in the last two weeks.

Supplementary Table SA3 presents null model results. While for psychological complaints the ICC at the country-year level was slightly larger than the ICC at the country level, for somatic complaints the ICCs were of comparable size. Supplementary Table SA4 displays tests of linearity for the changes over time in both outcomes. Psychological complaints exhibited nonlinear trends, including quadratic (B = 0.09, 95% CI: 0.01–0.17) and cubic (B = 0.19, 95% CI: 0.15–0.23) changes. Similarly, somatic complaints showed significant quadratic (B = 0.26, 95% CI: 0.21–0.38) and cubic (B = 0.23, 95% CI: 0.24–0.30) trends, with the linear trend being nonsignificant, as depicted in Figure 1. These results imply that if we refer to changes over time in the remainder of the paper, the changes are likely nonlinear rather than linear.

### Changes Over Time in Social Inequalities in Adolescents’ Psychological and Somatic Complaints

**FIGURE 2:**
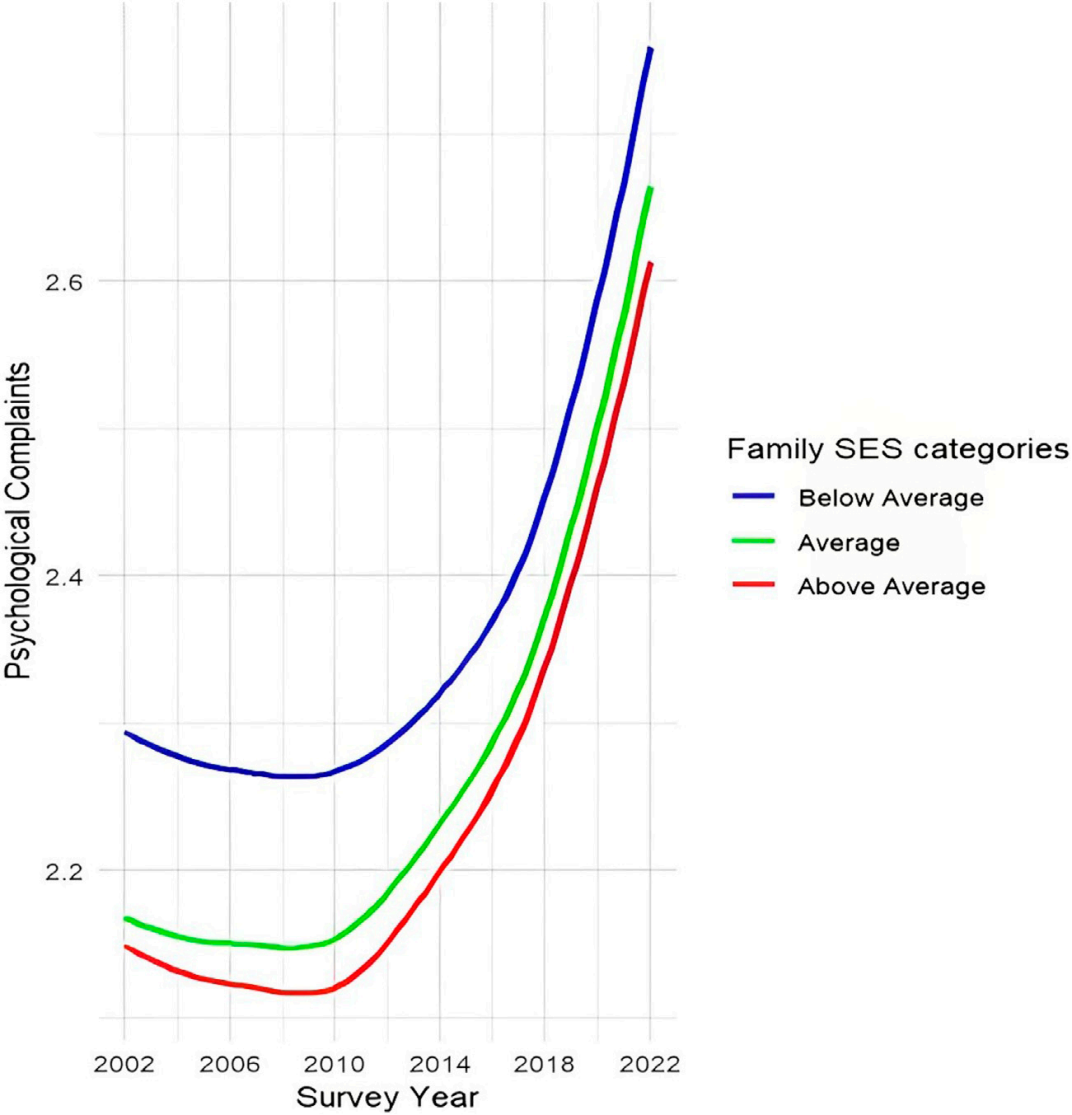
Trends in social inequalities in psychological complaints between 2002–2022 (Health Behaviour in School-aged Children, N_individuals_ = 902,682, N_countries_ = 32). Note: the scale ranges between 1 and 5 scores, with higher scores referring to more somatic or psychological symptoms in the last two weeks; three family SES categories are based on the mean and SD of the family SES: above average (mean + 1 SD or higher), average (within ± 1 SD of the mean) and below average (mean – 1 SD or lower).

**FIGURE 3:**
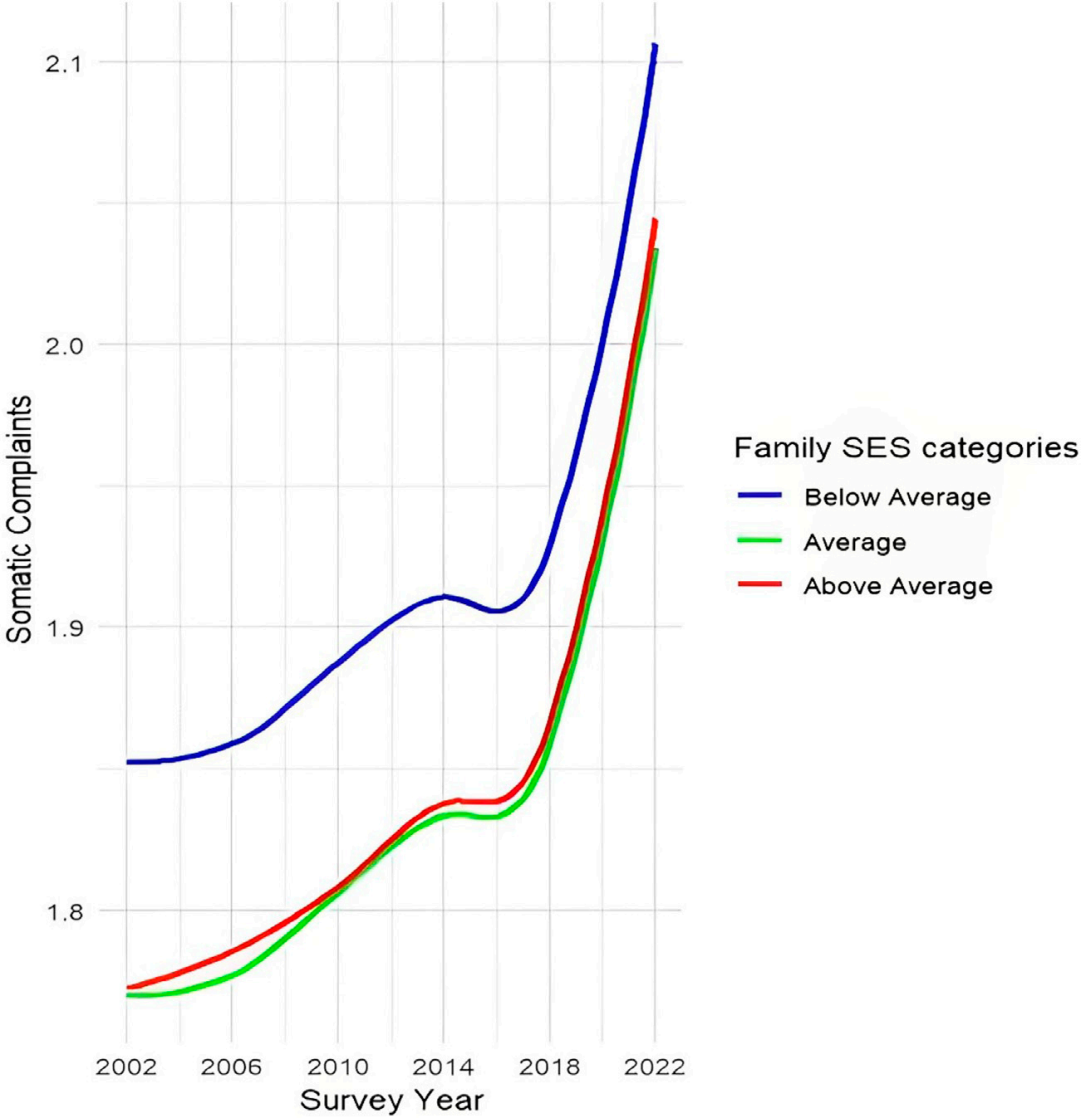
Trends in social inequalities in somatic complaints between 2002–2022 (Health Behaviour in School-aged Children, N_individuals_ = 902,682, N_countries_ = 32). Note: the scale ranges between 1 and 5 scores, with higher scores referring to more somatic or psychological symptoms in the last two weeks; three family SES categories are based on the mean and SD of the family SES: above average (mean + 1 SD or higher), average (within ± 1 SD of the mean) and below average (mean – 1 SD or lower).

Models 1 in Table 2 reveal a significant increase in both psychological (B = 0.09, 95% CI: 0.08–0.10) and somatic complaints (B = 0.05, 95% CI: 0.04–0.06) among adolescents over time. This observation aligns with the trends depicted in Figure 1, where a clear nonlinear increase in both types of complaints is visible. Family SES was negatively associated with psychological (B = −0.03, 95% CI: −0.04 to −0.02) and somatic complaints (B = −0.01, 95% CI: −0.02 to 0.00). The interaction term between family SES and time was not significant for either outcomes, implying that the negative association between family SES and psychological and somatic complaints remained stable over time on average across countries. This is also evident in Figures 2 and 3, showing a relatively constant disparity between the three family SES categories over time for both psychological and somatic complaints. Throughout the assessment period, adolescents with a below average family SES reported the most psychological and somatic complaints compared to their counterparts with average and above average family SES. A sharp increase in both psychological and somatic complaints occurred between 2018 and 2022, potentially indicating a COVID-19-related effect. To ensure the robustness of our findings, we excluded the most recent wave (i.e., 2022), which yielded similar results for Models 1 and 2c (Supplementary Table SA5).

**TABLE 2:** Results of multilevel models predicting psychological and somatic complaints between (Health Behaviour in School-aged Children, 2002–2022).

	Models 1		Models 2a		Models 2b		Models 2c	
	Psychological complaints*N*_*individuals*_ *= 902,682* *N*_*countries*_ *= 32*	Somatic complaints*N*_*individuals*_ *= 903,344* *N*_*countries*_ *= 32*	Psychological complaints*N*_*individuals*_ *= 902,682* *N*_*countries*_ *= 32*	Somatic complaints*N*_*individuals*_ *= 903,344* *N*_*countries*_ *= 32*	Psychological complaints*N*_*individuals*_ *= 891,218* *N*_*countries*_ *= 32*	Somatic complaints*N*_*individuals*_ *= 891,819* *N*_*countries*_ *= 32*	Psychological complaints*N*_*individuals*_ *= 507,787* *N*_*countries*_ *= 30*	Somatic complaints*N*_*individuals*_ *= 508,234* *N*_*countries*_ *= 30*
**Fixed effects**	Beta (95% CI)	Beta (95% CI)	Beta (95% CI)	Beta (95% CI)	Beta (95% CI)	Beta (95% CI)	Beta (95% CI)	Beta (95% CI)
Intercept	−0.52 (−0.59–0.45)***	−0.40 (−0.47–0.34)***	−0.51 (−0.58–−0.44)***	−0.40 (−0.46–−0.34)***	−0.44 (−0.51–−0.37)***	−0.34 (−0.40–−0.28)***	−0.76 (−1.01–−0.52)***	−0.38 (−0.61–−0.15)***
**Individual-level variables**								
Gender (*ref. = boys*)	0.35 (0.34–0.35)***	0.38 (0.38–0.39)***	0.35 (0.34–0.35)***	0.38 (0.38–0.39)***	0.29 (0.29–0.30)***	0.34 (0.34–0.35)***	0.38 (0.37–0.38)***	0.40 (0.39–0.40)***
Age	0.13 (0.13–0.13)***	0.14 (0.13–0.14)***	0.13 (0.13–0.13)***	0.14 (0.13–0.14)***	0.07 (0.07–0.07)***	0.09 (0.09–0.09)***	0.14 (0.13–0.14)***	0.14 (0.14–0.15)***
Family structure (*ref. = not living together with both parents*)	−0.07 (−0.08–−0.07)***	−0.07 (−0.07–−0.07)***	−0.07 (−0.08–−0.07)***	−0.07 (−0.07–−0.07)***	−0.07 (−0.07–−0.07)***	−0.07 (−0.07–−0.06)***	−0.08 (−0.08–−0.07)***	−0.07 (−0.07–−0.07)***
Family SES	−0.03 (−0.04 to −0.02)***	−0.01 (−0.02–0.00)**	−0.03 (−0.04 to −0.02)***	−0.01 (−0.02–0.00)**	−0.03 (−0.04 – - 0.02)***	−0.01 (−0.02–0.00)***	−0.07 (−0.12 to −0.01)***	−0.03 (−0.09–0.02)
Time	0.09 (0.08–0.10)***	0.05 (0.04–0.06)***	0.09 (−0.08–0.10)***	0.05 (0.04–0.06)***	0.08 (−0.06–0.09)***	0.04 (0.03–0.05)***	0.11 (0.06–0.17)***	0.02 (−0.03–0.07)
**Country-level variables**								
GNI	−0.03 (−0.08–0.02)	−0.01 (−0.06–0.03)	−0.03 (−0.09–0.02)	−0.02 (−0.07–0.03)	−0.03 (−0.08–0.01)	−0.01 (−0.06–0.04)	0.01 (−0.06–0.07)	0.02 (−0.05–0.09)
Social welfare	−0.05 (−0.09 to −0.01)*	−0.01 (−0.05–0.02)	−0.04 (−0.08–0.00)*	−0.01 (−0.04–0.02)	−0.05 (−0.08 to −0.02)*	−0.01 (-0.04–0.02)	−0.01 (−0.05–0.02)	−0.01 (−0.05–0.02)
Income inequality			−0.05 (−0.12–0.02)	−0.02 (−0.07–0.04)				
Mean income inequality			0.10 (0.01–0.18)*	0.05 (−0.02–0.00)				
Schoolwork pressure					0.19 (0.19–0.19)***	0.13 (0.13–0.14)***		
Mean schoolwork pressure					−0.04 (−0.09–0.01)	−0.03 (−0.08–0.02)		
Internet activity							−0.19 (−0.30 to −0.08)**	−0.15 (−0.26 to −0.05)*
Mean internet activity							0.00 (−0.07–0.06)	−0.02 (−0.09–0.04)
**Cross-level interactions**								
Family SES*time	0.00 (−0.00–0.00)	0.00 (−0.00–0.00)	0.00 (−0.00–0.00)	0.00 (−0.00–0.00)	0.00 (−0.01–0.01)	0.00 (−0.00–0.00)	0.01 (−0.00–0.02)	0.01 (−0.01–0.02)
Family SES*income inequality			−0.02 (−0.03 to −0.01)***	−0.01 (−0.02–0.00)*				
Time*income inequality			−0.01 (−0.02–0.01)	0.00 (−0.01–0.01)				
Family SES*income inequality*time			0.00 (0.00–0.01)*	0.01 (−0.00–0.00)				
Family SES*schoolwork pressure					−0.01 (−0.06 to −0.03)***	−0.01 (−0.01–0.00)**		
Time*schoolwork pressure					0.03 (0.03–0.03)***	0.02 (0.02–0.02)***		
Family SES*schoolwork pressure*time					0.00 (−0.00–0.00)	0.00 (−0.00–0.00)		
Family SES*internet activity							−0.01 (−0.04–0.02)	−0.01 (−0.05–0.02)
Time*internet activity							0.06 (0.04–0.07)***	0.05 (0.04–0.07)
Family SES*internet activity*time							−0.00 (−0.01–0.00)	0.00 (−0.00–0.01)
**Random effect**								
Family SES*time (slope)	0.02*	0.01*	0.02*	0.01*	0.02*	0.01*	0.01*	0.00*
**Model fit**								
AIC	2,518,675	2,211,851	2,518,694	2,211,885	2,395,363	2,136,670	1,439,264	1,264,086

*p < 0.05, **p < .01, ***p < .001. *Note*: Results are beta coefficient with 95% confidence interval (CI).

### Variations Between Countries in the Trends in Social Inequalities in Adolescents’ Psychological and Somatic Complaints

Models 1 in Table 2 showed significant random slopes suggesting country differences in trends in social inequalities for both psychological (*τ* = 0.02, p < 0.05) and somatic complaints (*τ* = 0.01, p < 0.05), as depicted in Supplementary Figures SA1 and SA2. Although most countries showed stable social inequalities over time (e.g., Netherlands and England), a few showed increasing (e.g., Canada and Austria) or decreasing social inequalities (e.g., Portugal and Lithuania).

**FIGURE 4:**
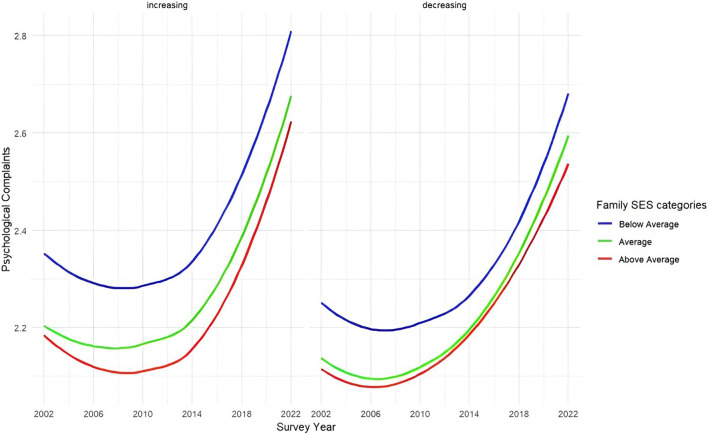
Trends in social inequalities of psychological complaints in countries with increasing *versus* decreasing income inequality (Health Behaviour in School-aged Children, N_individuals_ = 902,682, N_countries_ = 32). Note: the scale ranges between 1 and 5 scores, with higher scores referring to more somatic or psychological symptoms in the last two weeks; three family SES categories are based on the mean and SD of the family SES: above average (mean + 1 SD or higher), average (within ± 1 SD of the mean) and below average (mean – 1 SD or lower); Classification of countries as ‘decreasing’ or ‘increasing’ in income inequality was based on GINI values observed in 2002 and 2022; In 17 increasing income inequality countries the average Gini increased from 0.277 in 2002 to 0.293 in 2022, and in 15 decreasing income inequality countries the average Gini decreased from 0.308 in 2002 to 0.295 in 2022. Countries with increasing income inequality were: Austria, Denmark, Finland, France, Germany, Hungary, Italy, Iceland, Latvia, Lithuania, Netherlands, Norway, Slovenia, Slovakia, Spain, Sweden, and Switzerland. Countries with decreasing income inequality were: Belgium Flemish, Belgium French, Canada, Czech Republic, Estonia, Ireland, Israel, Greece, Poland, Portugal, England, Scotland, and Wales.

To explain these country differences, we tested the role of three societal changes. Considering country-level income inequality, Models 2a in Table 2 demonstrated that income inequality was not significantly associated with psychological and somatic complaints. The two-way interaction term between family SES and country-level income inequality was negatively associated with both psychological (B = −0.02, 95% CI: −0.03 to −0.01) and somatic complaints (B = −0.01, 95% CI: −0.02 to 0.00), suggesting that greater income inequality, whether from differences between countries or increases over time, was associated with greater social inequalities in psychological and somatic complaints. When excluding the three-way interactions, the results were similar (Supplementary Table SA6). To evaluate whether changes in income inequality within countries were linked to changes in social inequalities in psychological and somatic complaints, we examined three-way interactions (i.e., family SES*income inequality*time). This three-way interaction was significant for psychological complaints (B = 0.00, 95% CI: 0.00–0.01), indicating that in countries where income inequality increased over time, social inequalities in psychological complaints decreased, while social inequalities remained relatively stable in countries with decreasing income inequality. This trend is illustrated in Figure 4, with the division of countries listed below the figure.

Concerning schoolwork pressure, Models 2b in Table 2 demonstrated that schoolwork pressure was positively associated with psychological (B = 0.19, 95% CI: 0.19 to 0.19) and somatic complaints (B = 0.13, 95% CI: 0.13–0.14), implying that adolescents in countries or during periods characterized by higher levels of schoolwork pressure reported more psychological and somatic complaints. The two-way interaction term between schoolwork pressure and family SES was significant for both psychological (B = −0.01, 95% CI: −0.06 to 0.03) and somatic complaints (B = −0.01, 95% CI: −0.01 to 0.00), indicating that the negative association between family SES and psychological and somatic complaints was more pronounced in countries or during times characterized by higher levels of schoolwork pressure. Similar results were obtained when excluding the three-way interactions (Supplementary Table SA6). However, we did not detect a significant three-way interaction (i.e., family SES*schoolwork pressure*time) for either complaints. This means that although social inequalities in psychological and somatic complaints were more pronounced in countries or times with higher levels of schoolwork pressure, trends in social inequalities in psychological and somatic complaints were not related to national-level changes in schoolwork pressure.

Regarding internet activity, Models 2c in Table 2, showed that internet activity was negatively associated with both psychological (B = −0.19, 95% CI: −0.30 to −0.08) and somatic complaints (B = −0.15, 95% CI: −0.26 to −0.05). Notably, when excluding all interactions, internet activity was no longer significantly related to either type of complaint. For both outcomes, the two-way interaction term (i.e., family SES*internet activity) was not significant, suggesting that the association between family SES and psychological and somatic complaints did not vary with internet activity levels at the country level. When excluding only the three-way interactions, the two-way interaction term was only significant for psychological complaints (Supplementary Table SA6), indicating that the negative association between family SES and psychological complaints was more pronounced in countries or during times characterized by higher levels of internet activity. We did not detect a significant three-way interaction term (i.e., family SES*internet activity*time), implying that trends in social inequalities regarding these complaints were not associated with national-level changes in internet activity.

## Discussion

### Main Findings

Our study assessed trends in social inequalities in adolescents’ mental health problems from 2002 to 2022 across 32 European and North American countries and the role of country-level income inequality, schoolwork pressure, and internet activity. We observed a steep nonlinear increase in both psychological and somatic complaints across countries, specifically between 2018 and 2022. Adolescents from higher SES families consistently reported fewer psychological and somatic complaints across countries throughout the assessment period. This negative association between family SES and both types of complaints remained, on average across countries, stable over time. Yet, we observed some cross-national variation in these trends, with most countries maintaining stable social inequalities, while a few exhibited either increasing or decreasing social inequalities. Further, our results demonstrated that only income inequality accounted for this cross-national variation. As income inequality increased over time within countries, social inequalities in psychological complaints became smaller. Additionally, we found that social inequalities in psychological and somatic complaints were more pronounced in countries or times with greater income inequality or schoolwork pressure. Besides, more pronounced social inequalities in psychological complaints were observed in countries or times with higher levels of internet activity.

### Interpretation of the Findings

Our results highlighted that there is a nonlinear increase in both psychological and somatic complaints which may be attributed to the COVID pandemic and/or other factors such as an overinterpretation of mental health problems as suggested by the prevalence inflation hypothesis [46]. However, congruent with most prior studies examining earlier periods [14–16], our results further demonstrated stable social inequalities in these complaints on average across countries between 2002 and 2022. This suggests persistent social inequalities in adolescents’ mental health problems over time. Although we observed stable social inequalities in psychological and somatic complaints on average across countries, it is important to acknowledge the variability across countries in these trends, echoing Elgar et al. [9] and Moor et al. [16]. In most countries, stable social inequalities in psychological and somatic complaints were found, whereas a few displayed either increasing or decreasing social inequalities. Among the analyzed societal changes, only income inequality was able to explain some of the country-level differences in trends in social inequalities in psychological complaints. Our results indicated that increases in income inequality within countries over time were linked to smaller social inequalities in psychological complaints over time. This counterintuitive result might be explained by the initially greater social inequalities in psychological complaints in countries experiencing increases over time in income inequality, which may have allowed for more scope for reducing social inequalities. Notably, towards the end of the study period, social inequalities seemed to converge between countries with increasing and decreasing income inequality. An alternative explanation for our finding may be that other variables not captured in our study explain why we found decreasing trends in social inequalities in psychological complaints in countries with increasing national-level income inequality. The group of countries experiencing increases in income inequality includes, among other countries, all Scandinavian countries which are known for their social democratic systems, extensive family policies, and universal healthcare [47, 48]. These country characteristics might have contributed to the observed effect of income inequality.

Besides focusing on changes in social inequality trends within countries over time, we also looked at either between countries or over time by assessing to what extent income inequality, schoolwork pressure, and internet activity are related to social inequalities in psychological and somatic complaints. Consistent with previous studies investigating the role of country-level income inequality [49, 50], we observed greater social inequalities in psychological and somatic complaints with higher levels of income inequality, either from differences between countries or increases over time. While previous studies have not investigated the role of country-level schoolwork pressure, our findings align with Högberg [6] suggesting that social inequalities in mental health problems are stronger in countries with higher levels of schoolwork pressure. Both findings support the notion of cumulative disadvantage [19], where the combination of family-related stressors (e.g., parental conflict) [2] and stressors stemming from societal changes (e.g., schoolwork pressure and income inequality) can overwhelm adolescents [9]. For adolescents from low SES families (often facing more family-related stressors), living in countries characterized by higher levels of schoolwork pressure may exacerbate mental health problems, thus widening social inequalities in psychological and somatic complaints. Indeed, social comparison processes triggered by income inequalities and a competitive school context might interact to negatively impact adolescents’ mental health problems.

Our results for internet activity were mixed. We observed that social inequalities in somatic complaints were unrelated to internet activity. This may be attributed to the widespread availability of the internet, suggesting that internet activity does not differentiate between SES groups for somatic complaints. Our results for psychological complaints supported the notion of cumulative disadvantage, showing greater social inequalities concerning adolescents’ psychological complaints in countries or times with higher levels of internet activity [10]. One possible explanation for why we observe it for psychological complaints and not for somatic complaints is that these social comparisons being made online foster feelings of inferiority, which are primarily psychological in nature [51]. These feelings are more closely linked to psychological distress, such as anxiety and depression [52], rather than to somatic symptoms. While research on internet activity’s role in adolescent mental health inequalities is limited, Boer et al. [19] found that increases in country-level internet activity were associated with increases in country-level psychological complaints among adolescents. Our study expands on this finding, suggesting that these increases in internet activity are particularly harmful to adolescents from lower SES families. However, further research is needed to better understand the interaction between family affluence and internet activity and why associations may be different for psychological than for somatic complaints by for example testing relative inequalities in these complaints.

### Strengths and Limitations

A main strength of this study is its large, nationally representative sample, identical recruitment, and similar measurements over time and across countries which allowed us to explore cross-national trends. Additionally, we utilized validated scales for psychological and somatic complaints [24, 25]. Alongside these strengths, some limitations must be acknowledged. First, our repeated cross-sectional design hinders causal inference. For example, the social selection hypothesis suggests that reverse causality between family SES and psychological and somatic complaints is possible, meaning adolescents with more mental health problems might end up with lower SES due to the financial burden of their conditions (e.g., treatment costs) [53]. However, evidence from longitudinal studies indicates that the direction we tested is more likely than *vice versa* [3]. Second, we used internet activity as a proxy for social media use which may not fully capture the online behaviour we intended to assess, like online social comparisons [54] Additionally, the non-parallel survey cycles of HBSC and PISA may limit the accuracy of this proxy. Lastly, while we focused on three societal changes, other country-level processes could also alter trends in social inequalities in adolescents’ mental health problems, such as over-burdened health services that create greater barriers to care for lower SES groups, and shifts in family dynamics, including the rise of single-parent households or changes in parental involvement. Investigating other country-level variables and their role on these trends warrants further research.

### Conclusion

This study offers new insights into trends in social inequalities in adolescents’ mental health problems by examining societal changes. We observed a nonlinear increase in both psychological and somatic complaints over time across countries, with consistently higher complaints among adolescents from lower SES families. Stable trends in social inequalities for both outcomes were observed across countries, but these trends varied between countries. Only country-level income inequality may account for this cross-national variation, particularly concerning psychological complaints. Our findings highlight the persistence of social inequalities in adolescents’ mental health problems, indicating that current policies may not adequately address these disparities, necessitating intensified efforts and ongoing monitoring.
