# Supplementary material for: Social Inequalities in Adolescents’ Psychological and Somatic Complaints: Cross-National Trends Between 2002 and 2022 and the Role of Societal Changes
Source: Int J Public Health. 2025 Jan 31;69:1607709. doi: 10.3389/ijph.2024.1607709 (PMC11825262; doi:10.3389/ijph.2024.1607709)
Supplement: Supplementary file 1 [file DataSheet1.docx]

| **Table A1. Bivariate correlations between individual-level and country-level variables from 2002-2022 (N_individuals_ = 956,111, N_countries_ = 33)** | | | | | | | |
| --- | --- | --- | --- | --- | --- | --- | --- |
|  | **Independent variables** | | | | | **Outcome variables** | |
|  | 1 | 2 | 3 | 4 | 5 | Psychological complaints | Somatic complaints |
| **Individual-level variables** |  |  |  |  |  |  |  |
| 1. Gender (*ref. = boys*) | 1 |  |  |  |  | **0.173** | **0.189** |
| 2. Age | 0.001 | 1 |  |  |  | **0.141** | **0.143** |
| 3. Family structure (*ref. = not living together with both parents*) | **-0.002** | **-0.023** | 1 |  |  | **-0.108** | **-0.094** |
| 4. Family SES | **-0.043** | -0.002 | **0.123** | 1 |  | **-0.045** | **-0.028** |
| **Country-level variables** |  |  |  |  |  |  |  |
| 1. Income inequality | 1 |  |  |  |  | **0.181** | **0.183** |
| 2. Schoolwork pressure | **0.305** | 1 |  |  |  | **0.401** | **0.260** |
| 3. Internet activity | 0.006 | **0.436** | 1 |  |  | **0.686** | **0.508** |
| 4. GNI | **0.229** | 0.063 | **0.203** | 1 |  | 0.013 | **0.153** |
| 5. Social welfare | **-0.338** | -0.081 | -0.010 | **0.283** | 1 | -0.008 | 0.051 |

*Note:* significant correlations (p <0.05) are set in **bold**

| **Table A2. Variance Inflation Factor (VIF) of the independent variables under study when predicting psychological and somatic complaints** | | |
| --- | --- | --- |
|  | **Outcome variables** | |
|  | Psychological complaints | Somatic complaints |
|  | *N_individuals_ = 507,787* | *N_individuals_ = 508,234* |
|  | *N_countries_ =30* | *N_countries_ =30* |
| **Individual-level variables** | |  |
| Gender (*ref. = boys*) | 1.00 | 1.00 |
| Age | 1.00 | 1.00 |
| Family structure (*ref. = not living together with both parents*) | 1.03 | 1.03 |
| Family SES | 1.03 | 1.03 |
| Time | 7.60 | 7.60 |
| **Country-level variables** |  |  |
| GNI | 1.67 | 1.67 |
| Social welfare | 1.16 | 1.16 |
| Income inequality | 1.30 | 1.30 |
| Schoolwork pressure | 1.54 | 1.54 |
| Internet activity | 7.95 | 7.95 |

| **Table A3. Models 0 for predicting psychological and somatic complaints between 2002-2022** | | |
| --- | --- | --- |
|  | **Outcome variables** | |
|  | Psychological complaints | Somatic complaints |
|  | *N_individuals_ = 902,682* | *N_individuals_ = 903,344* |
|  | *N_countries_ = 32* | *N_countries_ = 32* |
| **Fixed effects estimates** | Beta (95% CI) | Beta (95% CI) |
| Intercept | 2.30 (2.25-2.35) | 1.85 (1.80 - 1.89) |
| **Random effect estimates** | |  |
| Countries | 0.02 | 0.02 |
| Country-year | 0.05 | 0.02 |
| Individual | 1.03 | 0.73 |
| **Intraclass correlation coefficients** | |  |
| Countries | 1.82% | 2.60% |
| Country-year | 4.55% | 2.60% |

*Note:* Results are beta coefficient with 95% confidence interval (CI)

| **Table A4. Assessment of linearity in the association between family SES and psychological and somatic complaints between 2002 and 2022** | | |
| --- | --- | --- |
|  | **Outcome variables** | |
|  | Psychological complaints | Somatic complaints |
|  | *N_individuals_ = 902,682* | *N_individuals_ = 903,344* |
|  | *N_countries_ =32* | *N_countries_ =32* |
| **Fixed effects estimates** | Beta (95% CI) | Beta (95% CI) |
| Intercept | -0.19 (1.07 – 1.20)*** | -0.30 (-0.36 – -0.24)*** |
| **Individual-level variables** | |  |
| Gender (*ref. = boys*) | 0.30 (0.29 – 0.30)*** | 0.38 (0.38 – 0.39)*** |
| Age | 0.13 (0.08 – 0.08)*** | 0.14 (0.13 – 0.14*** |
| Family structure (*ref. = not living together with both parents*) | -0.07 (-0.17 – -0.16)*** | -0.07 (-0.07 – -0.07)*** |
| Family SES | -0.03 (-0.11 – - 0.07)*** | -0.01 (-0.01 – 0.00)*** |
| **Country-level variables** |  |  |
| GNI | -0.01 (-0.05 – 0.04) | -0.01 (-0.05 – 0.03) |
| Social welfare | -0.03 (-0.06 – 0.01) | -0.01(-0.03 – 0.02) |
| **Linearity measures** |  |  |
| ns(time,3)1^a^ | -0.02 (-0.08 – 0.05) | -0.03 (-0.08 – 0.01) |
| ns(time,3)2^b^ | 0.09 (0.01 – 0.17)** | 0.26 (0.21 – 0.38)*** |
| ns(time,3)3^c^ | 0.19 (0.15 – 0.23)*** | 0.23 (0.24 – 0.30)*** |

*p < 0.05, **p < .01, ***p < .001. *Note*: Results are beta coefficient with 95% confidence interval (CI); ^a^ ns() denotes the linear spline; ^b^ ns() denotes the quadratic spline; ^c^ ns() denotes the cubic spline.

| **Table A5. Sensitivity analysis Models 1 and 2c when excluding wave 2022** | | | | |
| --- | --- | --- | --- | --- |
|  | **Models 1** | | **Models 2c** | |
|  | *Psychological complaints* | *Somatic complaints* | *Psychological complaints* | *Somatic complaints* |
|  | *N_individuals_ = 752,456* | *N_individuals_ = 753,138* | *N_individuals_ = 381,706* | *N_individuals_ = 382,488* |
|  | *N_countries_ =32* | *N_countries_ =32* | *N_countries_ =30* | *N_countries_ =30* |
| **Fixed effects** | B (95% CI) | B ( 95% CI) | B ( 95% CI) | B ( 95% CI) |
| Intercept | 1.30 (1.14-1.45)*** | 0.83 (0.72 – 0.93)*** | -2.43 (-6.40 – 1.53) | -0.55 (-3.29 – 2.20) |
| **Individual-level variables** |  |  |  |  |
| Gender (*ref. = boys*) | 0.30 (0.30-0.30)*** | 0.29 (0.28 – 0.29)*** | 0.33 (0.32 – 0.33)*** | 0.30 (0.29 – 0.30)*** |
| Age | 0.08 (0.08 – 0.08)*** | 0.06 (0.06 – 0.07)*** | 0.09 (0.08 – 0.09)*** | 0.07 (0.07 – 0.07)*** |
| Family structure (*ref. = not living together with both parents*) | -0.17 (-0.17 – -0.16)*** | -0.13 (-0.13 – -0.13)*** | -0.18 (-0.18 – -0.17)*** | -0.13 (-0.14 – -0.13)*** |
| Family SES | -0.09 (-0.13 – -0.05)*** | -0.03 (-0.05 – -0.01)** | 0.66 (-1.66 – 2.98) | 0.20 (-1.75 – 2.15) |
| Time | 0.23 (0.03 – 0.44)*** | 0.08 (0.00 – 0.15)** | 2.16 (-0.70 – 5.03)* | 2.72 (-2.33 – 7.77) |
| **Country-level variables** |  |  |  |  |
| GNI | -0.05 (-0.49 – 0.39) | -0.11 (-0.47 – 0.26) | 0.13 (-0.59 – 0.86) | 0.05 (-0.57 – 0.68) |
| Social welfare | -0.68 (-1.37 – 0.02) | 0.23 (-0.25 – 0.71) | -0.35 (-1.40 – 0.65) | 0.01 (-0.80 – 0.82) |
| Internet activity |  |  | 1.13 (0.01 – 2.25)* | 0.37 (-0.40 – 1.14) |
| Mean internet activity |  |  | 0.04 (-0.10 – 0.18) | -0.01 (-0.13 – 0.11) |
| **Cross-level interactions** |  |  |  |  |
| Family SES*time | -0.06 (-0.28 – 0.15) | 0.01 (-0.08 – 0.10) | -0.67 (-1.54 – 0.20) | -0.38 (-1.79 – 1.03) |
| Family SES*internet activity |  |  | -0.24 (-0.90 – 0.42) | -0.09 (-0.65 – 0.46) |
| Time*internet activity |  |  | 2.12 (-4.20 – -0.04)* | 0.74 (-2.17 – 0.68) |
| Family SES*internet activity*time |  |  | 0.43 (-0.79 – 1.66) | 0.15 (-0.88 – 1.18) |
| **Random effects** |  |  |  |  |
| Family SES*time (slope) | 0.03* | 0.06* | 0.01* | 0.01* |

*p < 0.05, **p < .01, ***p < .001. *Note*: Results are beta coefficient with 95% confidence interval (CI)

| **Table A6. Results of multilevel models predicting psychological and somatic complaints between 2002-2022 excluding the three-way interactions** | | | | | | | | |
| --- | --- | --- | --- | --- | --- | --- | --- | --- |
|  | **Models 2a** | | **Models 2b** | | | **Models 2c** | | |
|  | Psychological complaints | Somatic complaints | Psychological complaints | Somatic complaints | | Psychological complaints | | Somatic complaints |
|  | *N_individuals_ = 902,682* | *N_individuals_ = 903,344* | *N_individuals_ = 891,218* | *N_individuals_ = 891,819* | | *N_individuals_ = 507,787* | | *N_individuals_ = 508,234* |
|  | *N_countries_ =32* | *N_countries_ =32* | *N_countries_ =32* | *N_countries_ =32* | | *N_countries_ =30* | | *N_countries_ =30* |
| **Fixed effects** | Beta (95% CI) | Beta (95% CI) | Beta (95% CI) | Beta (95% CI) | | Beta (95% CI) | | Beta (95% CI) |
| Intercept | 1.07 (1.00 0 1.14)*** | 0.76 (0.70 – 0.81)*** | 1.62 (1.55 – 1.69)*** | 1.09 (1.03 – 1.14)*** | | 0.47 (-0.36 – 1.30)*** | | -0.28 (-0.87 – 0.31)*** |
| **Individual-level variables** | | |  | | |  | | |
| Gender (*ref. = boys*) | 0.36 (0.36 – 0.37)*** | 0.33 (0.33 – 0.33)*** | 0.31 (0.30 – 0.31)*** | 0.30 (0.29 – 0.30)*** | | 0.40 (0.40 – 0.41)*** | | 0.35 (0.35 – 0.36)*** |
| Age | 0.08 (0.08 – 0.08)*** | 0.07 (0.07 – 0.07)*** | 0.04 (0.04 -0.05)*** | 0.05 (0.05 – 0.05)*** | | 0.09 (0.09 – 0.09)*** | | 0.08 (0.08 – 0.08)*** |
| Family structure (*ref. = not living together with both parents*) | -0.18 (-0.18 – -0.17)*** | -0.14 (-0.14 – -0.14)*** | -0.16 (-0.16 – -0.16)*** | -0.13 (-0.13 – -0.12)*** | | -0.19 (-0.20 – -0.18)*** | | -0.15 (-0.15 – -0.14)*** |
| Family SES | -0.10 (-0.13 – -0.06)*** | -0.04 (-0.06 – 0.01)** | -0.10 (-0.14 – -0.07)*** | -0.04 (-0.06 – -0.01)** | | -0.27 (-0.48 – -0.06)*** | | -0.10 (-0.28 – -0.08) |
| Time | 0.32 (0.22 – 0.42)*** | 0.25 (0.17 – 0.33)*** | 0.28 (0.18 – 0.38)*** | 0.22 (0.14 – 0.20)*** | | 1.29 (-0.33 – 2.90)*** | | 0.64 (0.21 – 1.06)*** |
| **Country-level variables** | | |  | | |  | | |
| GNI | 0.00 (-0.04 – 0.05) | -0.01 (-0.05 – 0.03) | 0.00 (-0.04 – 0.05) | 0.00 (-0.04 – -0.03) | | 0.02 (-0.06 - 0.09) | | 0.02 (-0.04 – 0.08) |
| Social welfare | -0.03 (-0.06 – 0.00) | 0.00 (-0.03 – 0.02) | -0.03 (-0.06 – 0.00)* | -0.01 (-0.03 – 0.02) | | -0.02 (-0.06 – -0.02) | | -0.02 (-0.05 – 0.01) |
| Income inequality | 0.02 (-0.03 – 0.07) | 0.01 (-0.03 – 0.05) |  |  | |  | |  |
| Mean income inequality | 0.04 (-0.03 – 0.11) | 0.03 (-0.03 – 0.08) |  |  | |  | |  |
| Schoolwork pressure |  |  | 0.22 (0.21 – 0.22)*** | 0.12 (0.12 – 0.13)*** | |  | |  |
| Mean schoolwork pressure |  |  | -0.05 (-0.11 – 0.01) | -0.03 (-0.08 – 0.01) | |  | |  |
| Internet activity |  |  |  |  | | -0.07 (-0.26 – 0.13) | | -0.11 (-0.25 – 0.03) |
| Mean internet activity |  |  |  |  | | 0.01 (-0.07 – 0.09) | | -0.01 (-0.07 – 0.06) |
| **Cross-level interactions** | | |  | |  |  |  | |
| Family SES*time | 0.00 (-0.01 – 0.01) | 0.00 (-0.00 – 0.01) | 0.00 (-0.01 – 0.01) | 0.00 (0.00 – 0.01) | | 0.04 (0.00 - 0.09) | | 0.02 (-0.02 – 0.06) |
| Family SES*income inequality | -0.03 (-0.05 – -0.02)*** | -0.02 (-0.03 – -0.01)*** |  |  | |  | |  |
| Time*income inequality | 0.00 (-0.01 –0.01) | 0.00 (-0.01 – 0.01) |  |  | |  | |  |
| Family SES*schoolwork pressure |  |  | -0.04 (-0.05 – -0.03)*** | -0.02 (-0.02 – -0.01)*** | |  | |  |
| Time*schoolwork pressure |  |  | 0.03 (0.03 – 0.03)*** | 0.02 (0.02 – 0.02)*** | |  | |  |
| Family SES*internet activity |  |  |  |  | | -0.06 (-0.12 – -0.01)* | | -0.02 (-0.06 – 0.03) |
| Time*internet activity |  |  |  |  | | 0.03 (-0.02 – 0.07) | | 0.03 (0.00 – 0.06) |
| **Random effect** |  |  |  |  | |  | |  |
| Family SES*time (slope) | 0.02* | 0.01* | 0.02* | 0.01* | | 0.02* | | 0.01* |

*p < 0.05, **p < .01, ***p < .001. *Note*: Results are beta coefficient with 95% confidence interval (CI);

**Figure A1. Cross-national differences in the trends of the association between family SES and psychological complaints across countries (N_individuals_ = 902,682; N_countries_ = 32)**

**
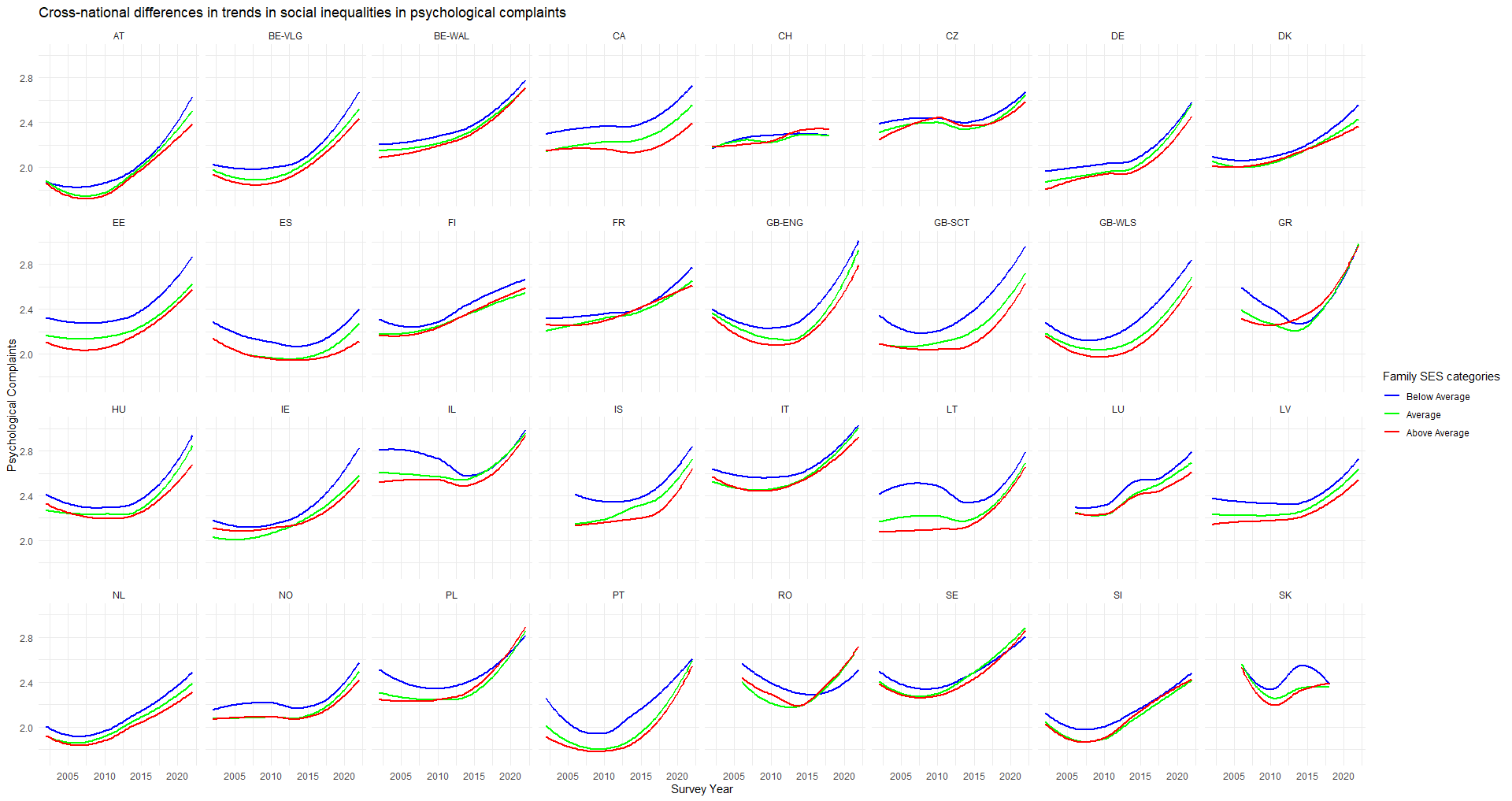
**

**Figure A2. Cross-national differences in the trends of the association between family SES and somatic complaints across countries (N_individuals_ =903,344; N_countries_ = 32)**

**
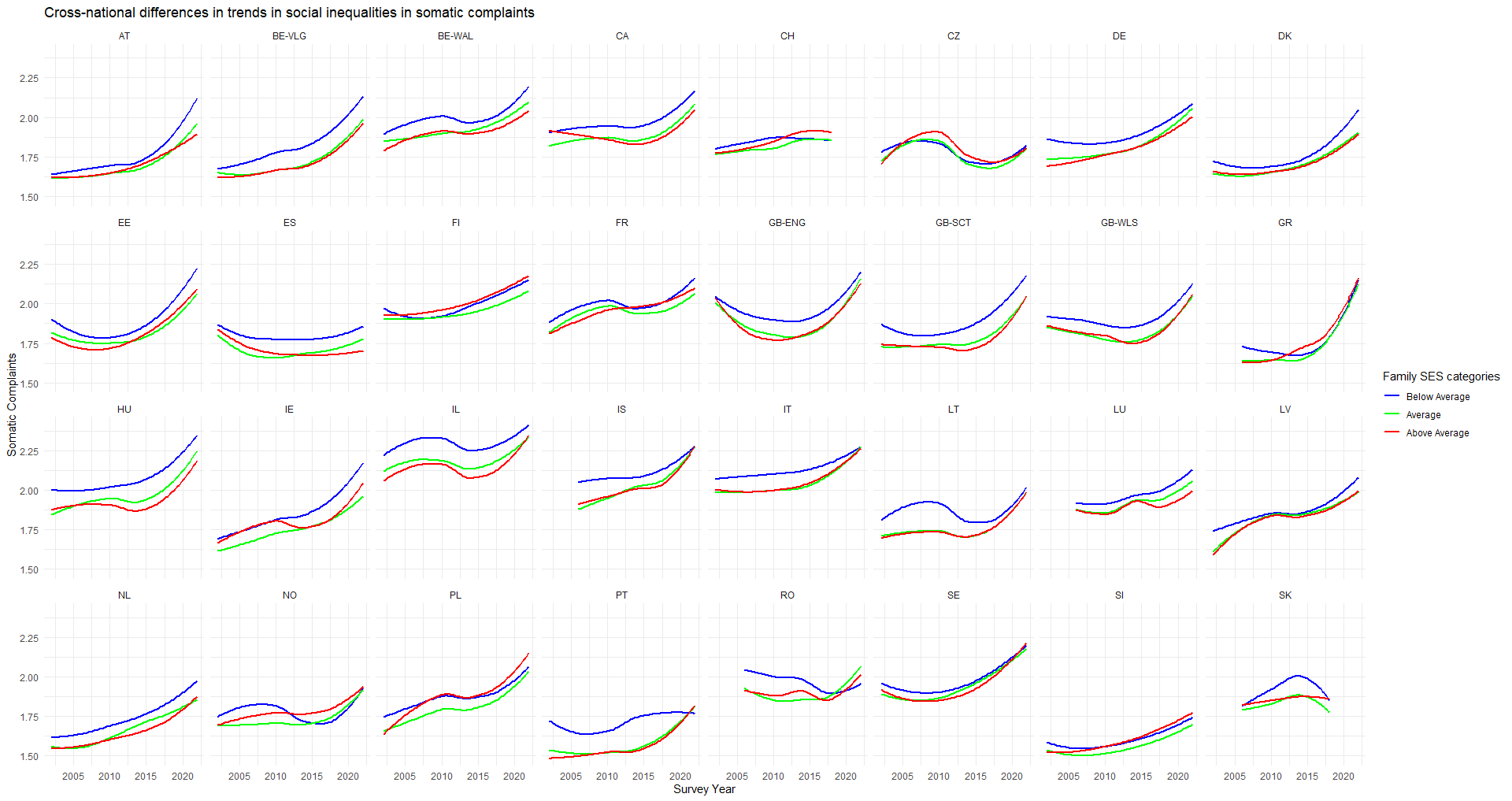
**
